# Metformin interferes with urinary creatinine measurement using enzymatic method

**DOI:** 10.1016/j.bbrep.2025.102264

**Published:** 2025-09-15

**Authors:** Akira Yoshimoto, Yoshifumi Morita, Yukio Kume, Naoyuki Yoshikawa, Yoshikazu Ono, Makoto Kurano, Yutaka Yatomi, Ryunosuke Ohkawa

**Affiliations:** a Department of Clinical Laboratory, The University of Tokyo Hospital, Tokyo, Japan; b Clinical Bioanalysis and Molecular Biology, Graduate School of Medical and Dental Sciences, Institute of Science Tokyo, Tokyo, Japan; c Department of Clinical Laboratory Medicine, Graduate School of Medicine, The University of Tokyo, Tokyo, Japan; d Graduate School, International University of Health and Welfare, Tokyo, Japan

## Abstract

Abnormal reaction curves were observed in urinary creatinine tests in patients with diabetes undergoing metformin therapy. Therefore, we investigated whether metformin interferes with urinary creatinine measurements.

First, the reaction curves of urinary creatinine measurements from 328 patients were analyzed, focusing on the maximum reaction speed, final reaction speed, and their ratio. Next, the reaction curves of the creatinine solution with and without metformin were analyzed. To elucidate the mechanism of metformin interference, solutions of creatinine, creatine, sarcosine, and hydrogen peroxide with and without metformin were analyzed using a one-reagent measurement in which reagents 1 and 2 were pre-mixed.

As the results, in the 84 patients taking metformin, the maximum reaction speed significantly decreased, whereas the final reaction speed and the ratio of the two speeds significantly increased (p < 0.001). Notably, the area under the curve of the ratio of the two speeds was 0.84 (95 % confidence interval: 0.78–0.89) for detecting metformin use, suggesting that metformin inhibits the series of reactions involved in creatinine measurement and that reaction curve analysis can identify metformin use. Similar results were obtained in experiments using metformin-containing creatinine solutions. Metformin did not interfere with the reactions involving sarcosine or hydrogen peroxide but did interfere with those involving creatinine and creatine, indicating that metformin inhibits creatinase activity.

In conclusion, metformin inhibits reactions involved in urinary creatinine measurement in urine samples, potentially leading to falsely low urinary creatinine values and an inaccurate assessment of kidney function in patients with diabetes.

## Highlights

Abnormal reaction curves were observed in routine urinary creatinine assay.
Metformin inhibits creatinase, resulting in false-low urinary creatinine values.
The inhibition of metformin may occur even at a low creatinine level.
Dilution of urine samples may be useful for decreasing the effect of metformin.

## Graphical abstract

**Figure:**
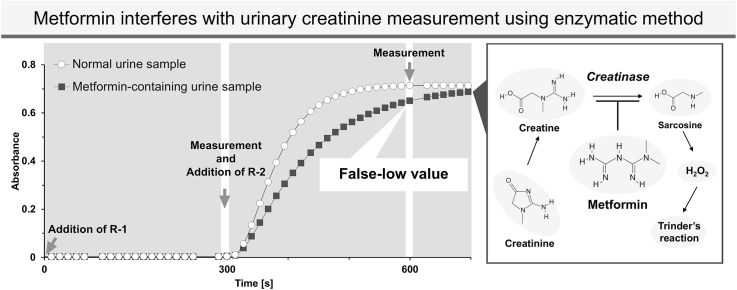

## 1 Introduction

Diabetes mellitus (DM) affected over 529 million individuals globally in 2021, with type 2 diabetes constituting 96 % of all cases [1]. Metformin, a first-line drug recommended by the World Health Organization [2,3], exerts its antihyperglycemic effects through multiple mechanisms, including decreased intestinal glucose absorption, enhanced peripheral glucose uptake, lowered fasting plasma insulin levels, and improved insulin sensitivity [3,4]. Beyond its antidiabetic properties, metformin has garnered significant attention for its potential benefits in various conditions such as cancer, aging, cardiovascular disease, and neurodegenerative disorders [4]. Metformin undergoes renal excretion in its unchanged form, with an elimination half-life of approximately 4–9 h in patients with normal renal function [3,5]. Urinary metformin concentrations range from 0 to 600 mg/dL [6,7]. Accurate assessment of renal function in patients with diabetes often involves measuring urinary biomarkers such as albumin. To precisely calculate biomarker concentrations, particularly the albumin-to-creatinine ratio, it is essential to accurately determine creatinine levels, which serve as a reference for urine concentration [8]. Although the Jaffe method has traditionally been used for creatinine determination, enzymatic methods have become more prevalent and are now recommended for the assessment of chronic kidney disease stages owing to their improved specificity and sensitivity [9,10]. Major enzymatic creatinine assay involves a series of five enzymatic reactions, culminating in the spectrophotometric measurement of the quinone chromogen formed by the reaction between hydrogen peroxide derived from creatinine and Trinder's reaction acceptor [11]. Although highly reliable, certain drugs, including dobutamine, dopamine, lidocaine, and homogentisic acid interfere with the accuracy of enzymatic creatinine measurements [[11], [12], [13]].

Autoanalyzers for biochemistry generate reaction curves for each specimen, enabling calculation of the measured values. Analyzing these reaction curves can be valuable for detecting potential interferences, such as M-proteins in bilirubin, urea, and high-density lipoprotein cholesterol measurements and homogentisic acid in creatinine measurement [[13], [14], [15], [16]]. During routine laboratory testing, unusual reaction curves were observed in the creatinine assay of urine specimens from patients with diabetes receiving metformin therapy. Given the structural similarity between metformin and creatine, we hypothesized that metformin interferes with enzymatic creatinine measurements. To the best of our knowledge, this potential interference has not been reported previously. Therefore, we aimed to investigate the effects of metformin on urinary creatinine levels.

## 2 Materials and methods

### 2.1 Study population

Inpatients and outpatients at the University of Tokyo Hospital between July and September 2020 were enrolled. Inclusion criterion required that patients had undergone all of the following clinical laboratory tests on the same day: blood tests including albumin, blood urea nitrogen (BUN), creatinine, aspartate aminotransferase (AST), alanine aminotransferase (ALT), total cholesterol, triglycerides, blood glucose, and hemoglobin A1c, as well as urinary tests including urinary creatinine, albumin/total protein, and dipstick tests. Patients with urinary creatinine levels greater than 250 mg/dL were excluded based on our institutional validation tests, which confirmed this value as the upper limit of linearity. A total of 328 patients were therefore enrolled in the analysis.

This study was conducted with the approval of the ethics committees of the University of Tokyo Hospital (approval number: 2019323NI) and the Institute of Science Tokyo (approval number: M2021-135) in compliance with the relevant guidelines and regulations. Patients participated in this study through an opt-out process. A full explanation of the study was publicly disclosed on the University of Tokyo Hospital webpage, and all patients were able to view this information at any time and had the freedom to decline participation. The content of the opt-out document on the website had been checked and approved by the ethics committee. The opt-out system is a commonly used approach for observational studies [[17], [18], [19], [20]].

### 2.2 Routine clinical laboratory tests in urine samples

Urinalysis was performed using an automatic clinical chemistry analyzer 7180 (Hitachi High-Tech Co., Tokyo, Japan) and an automatic urine strip analyzer US-3500 (Eiken Chemical Co., Tokyo, Japan).

### 2.3 Enzymatic measurement of urinary creatinine in patient specimens

Urinary creatinine levels were measured using L-Type Creatinine M (FUJIFILM Wako Pure Chemical Co., Osaka, Japan). The principle of this assay is illustrated in Supplemental Fig. 1. After endogenous creatine is eliminated in the 1st reaction, creatinine in the sample is converted to creatine by the action of creatininase in the 2nd reaction. The creatine formed is hydrolyzed by creatinase to product sarcosine and urea. The sarcosine produced is then decomposed by sarcosine oxidase to form hydrogen peroxide, glycine, and formaldehyde. In the presence of peroxidase, then hydrogen peroxide formed yields a blue pigment by quantitative oxidation condensation with 4-aminoantipyrine and N-(3-sulfopropyl)-3-methoxy-5-methylaniline (HMMPS). Creatinine concentration was calculated from the difference in absorbance of blue color at 600 nm after the 1st and 2nd reaction. Kinetic parameters, such as the maximum reaction speed, final reaction speed, and their ratio, were calculated from the reaction curves. A receiver operating characteristic (ROC) curve was generated using the ratio of the final reaction speed to the maximum reaction speed to determine whether patients took metformin or not.

### 2.4 Investigation of metformin interference with urinary creatinine measurement

BioMajesty JCA-BM6010 (JEOL Ltd., Tokyo, Japan) was used for the in vitro experiments to investigate the interference of metformin. The measurement procedures for both analyzers were performed according to the manufacturer's instructions. In the standard method, 2.5 μL of urine sample was mixed with 150 μL of reagent 1 and incubated for 5 min. Subsequently, 50 μL of reagent 2 was added, and the creatinine concentration was determined spectrophotometrically 5 min after.

To examine the effect of metformin on urinary creatinine levels, we conducted the following experiments. Creatinine solutions (50, 100, or 200 mg/dL) were prepared with and without various concentrations of metformin (100, 200, or 500 mg/dL). The solutions were then analyzed using standard methods. Kinetic parameters, such as the maximum reaction speed, final reaction speed, and their ratio, were calculated from the reaction curves.

### 2.5 Investigation of mechanism of metformin interference

To examine the effect of metformin on each enzyme reaction involved in creatinine measurement, we established the mixed method. In this method, the same volumes of sample and reagents were used; however, reagents 1 and 2 were premixed in the same ratio as in the standard method before being added to the sample. The mixed method can omit the elimination reactions of creatine, sarcosine, and hydrogen peroxide. Solutions of creatinine, creatine, sarcosine, and hydroperoxide (each at an equivalent molar concentration corresponding to creatinine 100 mg/dL) were measured using the mixed method, with and without metformin (200, 500, or 1000 mg/dL). To further investigate the kinetic effects, creatine solutions (20, 30, 50, 100, and 200 mg/dL) were analyzed using a mixed method, with and without metformin (100, 200, and 500 mg/dL). Lineweaver–Burk and Dixon plots were generated to assess the kinetic parameters and inhibition type of metformin [21].

To investigate the effect of metformin on creatininase activity, the following experiments were performed. The absorption spectra of creatinine (1 mg/dL), metformin (1 mg/dL), and the second reagent of the L-Type Creatinine M (final concentration 0.5 %) were measured. The maximum absorption wavelength of creatinine is 216 nm, which was selected as an indicator of creatinine concentration. Subsequently, to assess creatininase activity, 60 μL of sample, 2910 μL of phosphate buffered saline, and 30 μL of the second reagent (containing creatininase) were mixed and incubated at 37 °C. Decrease in absorbance at 216 nm was monitored using a UV-1280 spectrophotometer (Shimadzu, Kyoto, Japan). The rate of absorbance decrease per minute during the initial 3 min was defined as creatininase activity.

Finally, to examine the effect of preincubation with metformin on the extent of its interference, 100 mg/dL creatinine solutions with or without 100 mg/dL metformin were measured using both the standard and mixed methods. Each kinetic parameter was compared between the two methods.

### 2.6 Statistics

Statistical analyses were performed using R Studio version 2023.12.1 + 402 and R version 4.3.2. Wilcoxon rank sum test was used to compare the reaction rates between patients taking and not taking metformin. ROC curve analysis was performed to determine the optimal cut-off value for identifying patients receiving metformin therapy based on their medical records. The statistical power was 100 %, which was calculated from the patient numbers with and without metformin treatment, the area under the curve (AUC) value, and significant level 5 %. For experiments involving metformin, Tukey-Kramar test was used for multiple comparisons between creatinine solutions with and without metformin. Student's t-test was used to compare the effect of metformin on creatininase activity and the effect of metformin between standard method and mixed method. Statistical significance was set at a p-value of less than 0.05.

## 3 Results

### 3.1 Study population

Of the 328 patients whose urinary specimens were tested in routine laboratory tests, 213 had type 2 DM with or without metformin therapy (n = 84 and 129, respectively) (Supplemental Table 1). Patients with taking metformin showed almost same mean age; however, they seemed to be lower serum creatinine level than patients without taking metformin. In patients group taking metformin, GFR stage of almost patients were relatively low and patients group without metformin included relatively lower kidney function such as G4 and G5 stages than patients group with metformin.

### 3.2 Analysis of reaction curves of urinary creatinine in patients taking metformin

**Fig. 1:**
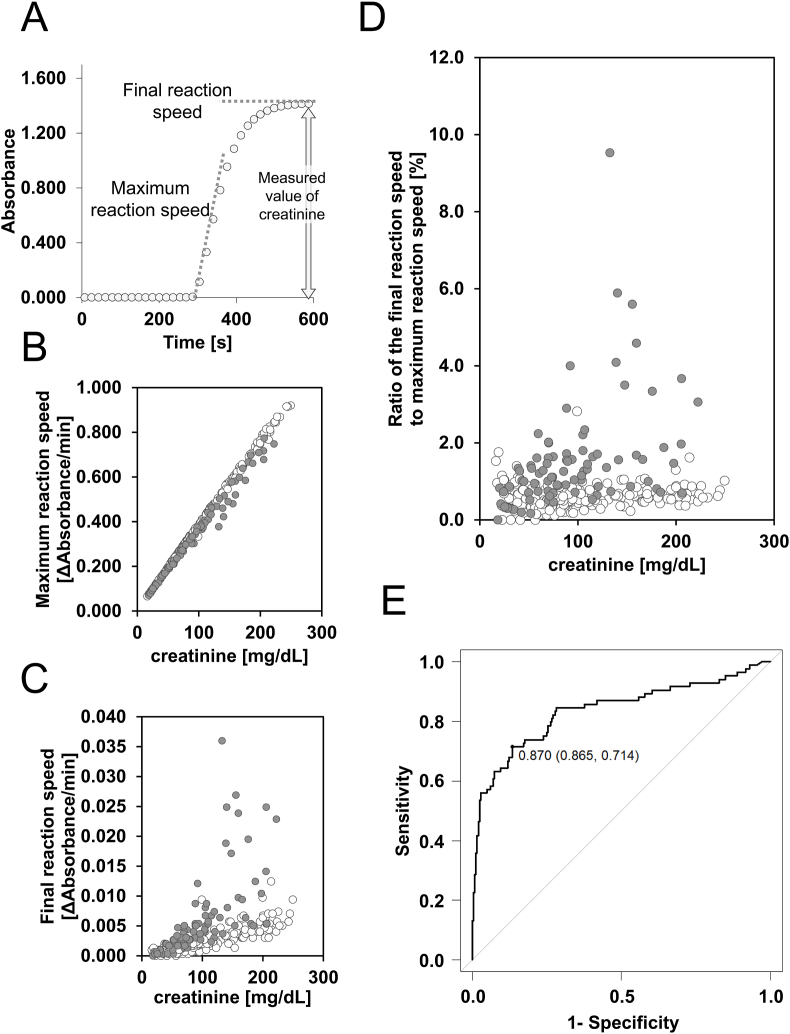
Analysis of urinary creatinine measurement in patients taking metformin. One example of reaction curve of urinary creatinine measurement and the maximum reaction speed and the final reaction speed were assessed (A). Relationship between the creatinine value and the maximum reaction speed (B), the final reaction speed (C), and the ratio of the final reaction speed to the maximum reaction speed (D). The black dots show 84 patients taking metformin and the white dots show 244 patients not taking metformin. Receiver operating characteristic curve analysis of the ratio of the final reaction speed to the maximum reaction speed against taking metformin (E).

Fig. 1A illustrates a typical reaction curve for the creatinine assay, indicating the maximum and final reaction rates. We analyzed the reaction curves of urinary creatinine measurements in 328 patients (244 metformin-untreated and 84 metformin-treated patients). There was no significant difference in urinary creatinine level between patients with and without taking metformin (88.4 [59.5–130.0] mg/dL vs. 80.6 [56.4–132.6] mg/dL, p = 0.661). However, compared to patients not taking metformin, those receiving metformin exhibited significantly lower maximum reaction speed (0.003705 [0.003581–0.003794] vs. 0.003877 [0.003836–0.003914], p < 0.001) and significantly higher final reaction speed (0.000047 [0.000029–0.000061] vs. 0.000022 [0.000016–0.000028], p < 0.001) per 1 mg/dL creatinine, despite having similar creatinine levels (Fig. 1B and C). These findings suggest that metformin may inhibit the enzymatic reactions involved in creatinine measurement, potentially leading to an underestimation of creatinine concentration. Furthermore, the ratio of the final reaction speed to the maximum reaction speed was significantly higher in patients administered metformin (Fig. 1D). The ROC curve analysis using the ratio of both speeds revealed that this ratio effectively discriminated between patients taking and not taking metformin, with an AUC of 0.84 (95 % confidence interval, 0.78–0.89) (Fig. 1E). An optimal cutoff value of 0.87 % yielded a sensitivity of 71 % and specificity of 87 % for identifying patients on metformin therapy.

### 3.3 Analysis of interference of metformin on urinary creatinine measurement

**Fig. 2:**
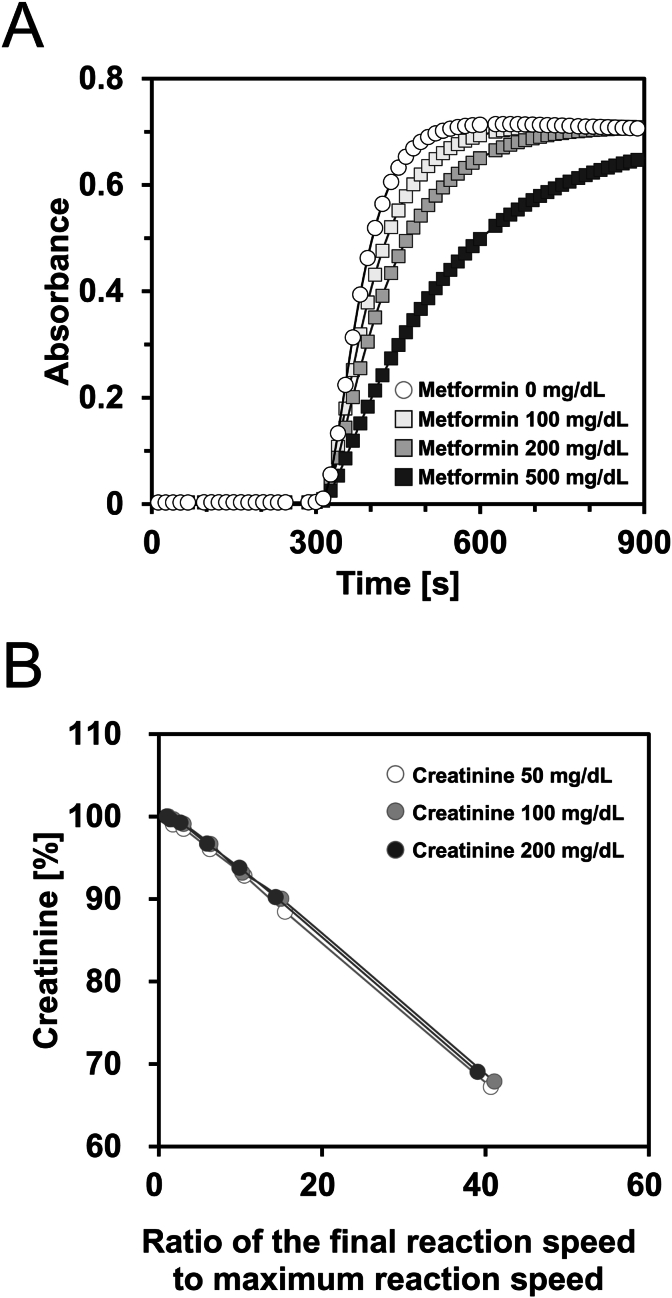
Effect of metformin on urinary creatinine measurement. Reaction curves of 100 mg/dL creatinine solution containing metformin (0, 100, 200, or 500 mg/dL) (A). Creatinine solutions (50, 100, or 200 mg/dL) containing metformin (0, 20, 50, 100, 150, 200, or 500 mg/dL) were measured. The lines show the relationship between the ratio of the final reaction speed to the maximum reaction speed and measured creatinine value assessed as percentage without metformin 100 % (B). n = 3.

To investigate the potential inhibitory effects of metformin on sequential creatinine measurement reactions, we examined the effect of varying metformin concentrations (100, 200, and 500 mg/dL) on creatinine levels (100 mg/dL). Metformin dramatically delayed the reaction kinetics of creatinine in a concentration-dependent manner (Fig. 2A). Analysis of the reaction curves revealed that metformin decreased the maximum reaction speed and increased the final reaction speed in a concentration-dependent manner (Table 1). Consequently, the ratio of the final-to-maximum reaction speed increased, and the measured creatinine values decreased with increasing metformin concentration. Notably, we observed a strong correlation between the increase in the ratio of both reaction speeds and the decrease in the measured creatinine values, regardless of the initial creatinine concentration (Fig. 2B). These results demonstrated that the ratio of both reaction speeds reflects the degree of inhibition by metformin.

**Table 1:** Analysis of creatinine reaction curves.

		Creatinine											
		50 mg/dL			p	100 mg/dL			p	200 mg/dL			p
Maximum reaction speed (ΔABS/min)													
Metformin	0	0.2038	±	0.0002		0.4025	±	0.0001		0.8000	±	0.0012	
(mg/mL)	100	0.1605	±	0.0005	<0.001	0.3194	±	0.0008	<0.001	0.6355	±	0.0016	<0.001
	200	0.1239	±	0.0001	<0.001	0.2503	±	0.0006	<0.001	0.4980	±	0.0008	<0.001
	500	0.0719	±	0.0004	<0.001	0.1435	±	0.0003	<0.001	0.2893	±	0.0004	<0.001
Final reaction speed (ΔABS/min)													
Metformin	0	0.0024	±	0.0001		0.0046	±	0.0004		0.0077	±	0.0028	
(mg/mL)	100	0.0100	±	0.0002	<0.001	0.0202	±	0.0005	<0.001	0.0376	±	0.0019	<0.001
	200	0.0192	±	0.0002	<0.001	0.0375	±	0.0002	<0.001	0.0713	±	0.0008	<0.001
	500	0.0292	±	0.0002	<0.001	0.0590	±	0.0005	<0.001	0.1129	±	0.0008	<0.001
The ratio of the final reaction speed to maximum reaction speed (%)													
Metformin	0	1.2	±	0.1		1.1	±	0.1		1.0	±	0.3	
(mg/mL)	100	6.3	±	0.1	<0.001	6.3	±	0.1	<0.001	5.9	±	0.3	<0.05
	200	15.5	±	0.1	<0.001	15.0	±	0.1	<0.001	14.3	±	0.1	<0.01
	500	40.7	±	0.4	<0.001	41.1	±	0.3	<0.001	39.0	±	0.3	<0.01
Measured value of creatinine (mg/dL)													
Metformin	0	53.4	±	0.0		105.7	±	0.1		208.5	±	0.3	
(mg/mL)	100	51.3	±	0.2	<0.001	102.2	±	0.2	<0.001	201.7	±	0.5	<0.001
	200	47.3	±	0.0	<0.001	95.2	±	0.2	<0.001	188.2	±	0.2	<0.001
	500	35.9	±	0.1	<0.001	71.7	±	0.1	<0.001	144.0	±	0.1	<0.001

Each p-value was calculated using the Tukey Kramar test vs. metformin 0 mg/dL.

### 3.4 Mechanism of metformin interference

**Fig. 3:**
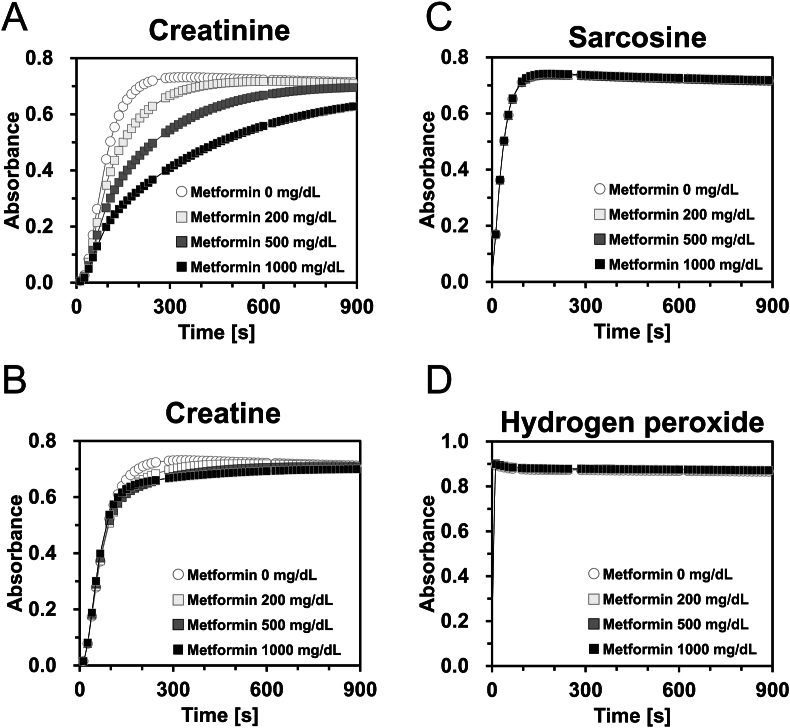
Analysis of the metformin-inhibition mechanism of creatinine measurement. Creatinine (A), creatine (B), sarcosine (C), or hydroperoxides (D) solution equivalent to 100 mg/dL of creatinine was measured by mixed method with and without metformin (200, 500, or 1000 mg/dL). n = 3.

**Fig. 4:**
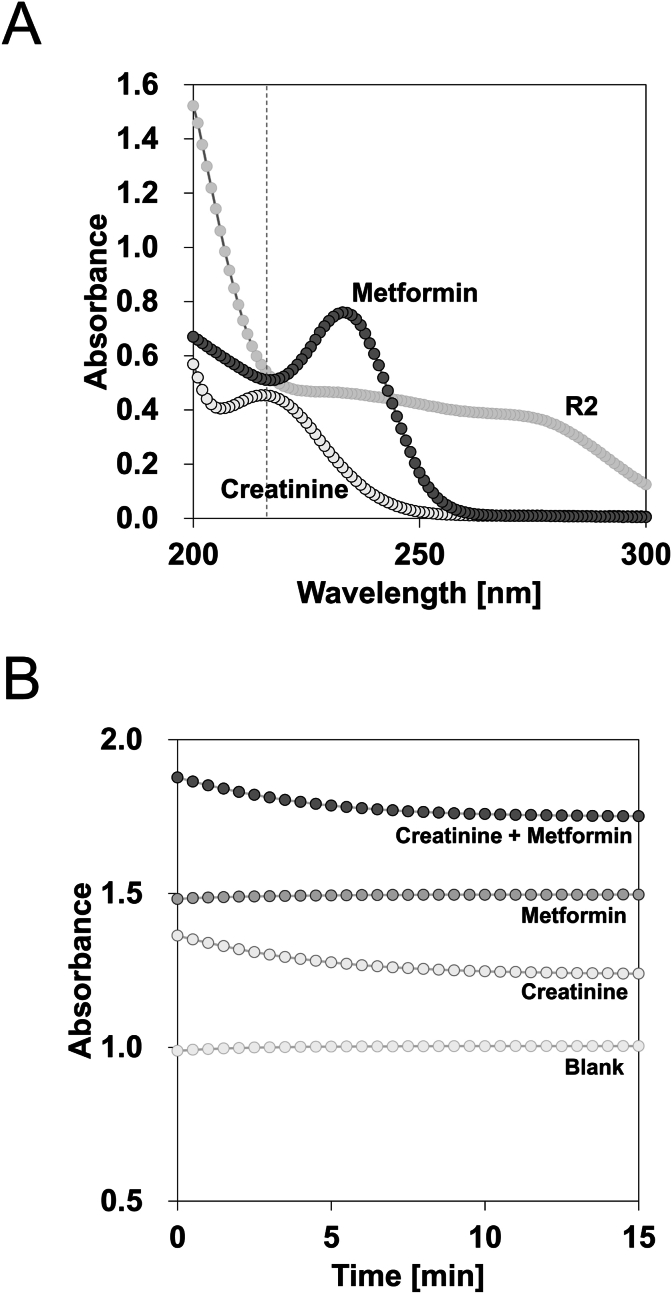
Effect of metformin on creatininase reaction. Absorption spectrum of creatinine (1 mg/dL), metformin (1 mg/dL), and creatinine measurement reagent 2 (R2) (Final 0.5 %) (A). The dot line shows 216 nm which was selected as an indicator of creatinine. One mg/dL of creatinine was added to R2 (Final 1 %) with and without metformin (1 mg/dL), and the absorbance was monitored (B). n = 3.

To identify the specific enzymatic reaction steps inhibited by metformin, we examined the effect of metformin on creatinine, creatine, sarcosine, and hydrogen peroxide levels using a mixed method that preserved endogenous creatine. Metformin significantly inhibited reactions involving creatinine and creatine as substrates (Fig. 3A and B); however, it had no effect on reactions involving sarcosine and hydrogen peroxide (Fig. 3C and D). These kinetic parameters were also analyzed (Supplementary Table 2). These results suggest that metformin primarily inhibits creatinase activity. However, the effect of metformin on creatininase activity remains unclear because the reaction with creatinine involves both creatininase and creatinase. To further investigate the effects of metformin on creatininase activity, we examined the absorption spectra of creatinine, metformin, and the creatinine measurement reagent 2. Because the absorbance at 216 nm was the maximum absorption wavelength of creatinine (Fig. 4A), we monitored the absorbance at 216 nm with and without the addition of reagent 2, which contained creatininase. The absorbance decreased with the addition of reagent 2, indicating creatininase activity. However, the decomposition speed of creatinine was not significantly changed in the presence of metformin (0.024 ± 0.001 vs. 0.025 ± 0.001/min, respectively, *p* = 0.233)(Fig. 4B), suggesting that metformin does not directly inhibit creatininase activity under these conditions.

**Fig. 5:**
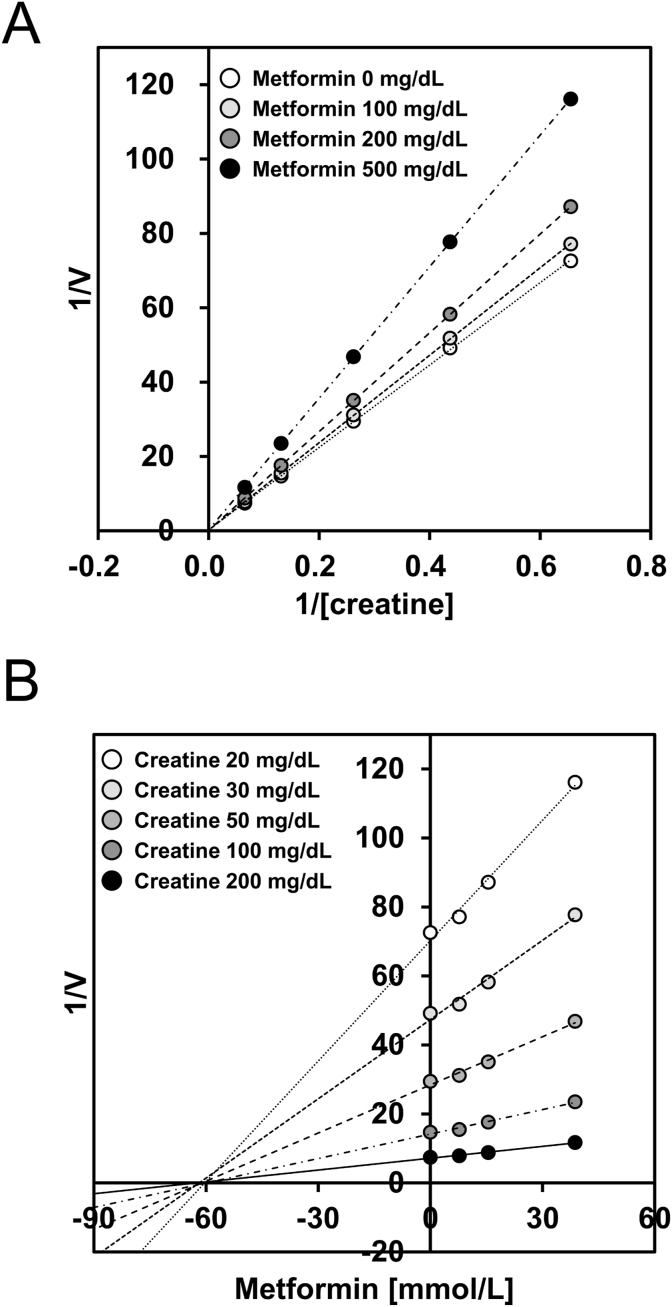
Analysis of metformin-inhibition type on creatinase. Creatine solution (20, 30, 50, 100, or 200 mg/dL) was measured by mixed method with and without metformin (100, 200, or 500 mg/dL). Lineweaver Burk-plot (A) and Dixon-plot (B). n = 3.

**Fig. 6:**
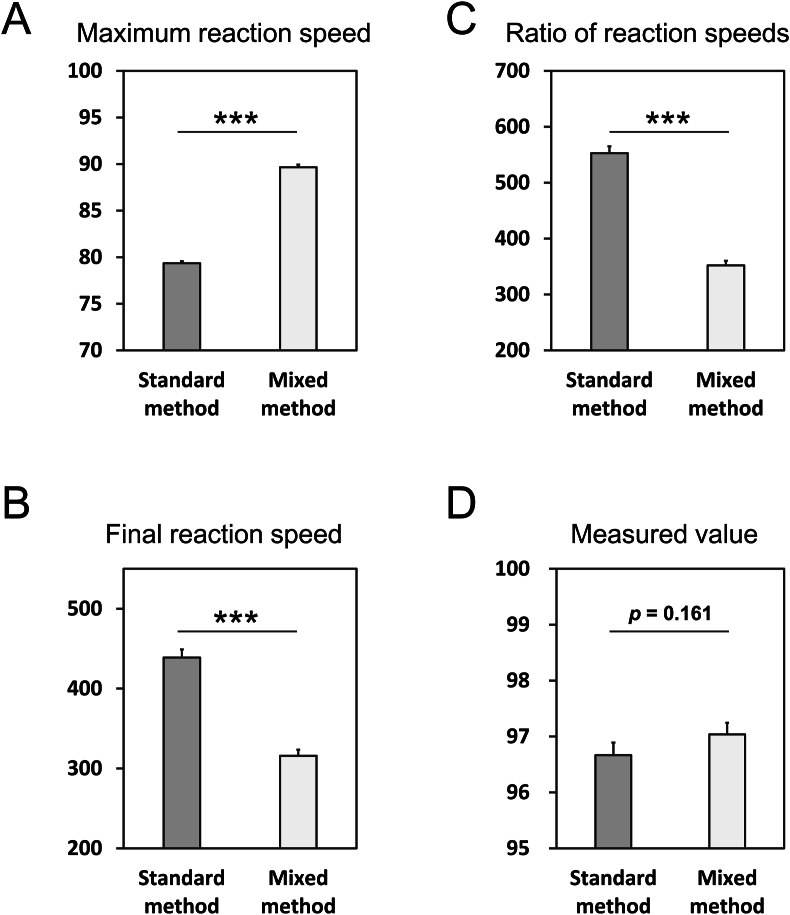
Comparison of degree of metformin-inhibition between the two and one reagent systems. Creatinine solutions (100 mg/dL) with or without 100 mg/dL of metformin were measured by the standard (dark gray bars) and mixed method (white bars). The maximum reaction speed (A), the final reaction speed (B), ratio of the final reaction speed to the maximum reaction speed (C), and measured value of creatinine (D) were expressed as the percentages calculated from 100 % of metformin 0 mg/dL, respectively. Each p-value was calculated using the student t-test. ∗∗∗p < 0.001. mean ± SD. n = 3.

Lineweaver-Burk and Dixon plots were generated to further characterize the type of inhibition (Fig. 5A and B). While the Lineweaver-Burk plot showed an apparent intersection near the origin, both plots demonstrated an intersection point on the x-axis, indicating that metformin inhibited creatinase via a non-competitive mechanism. Furthermore, the inhibitory effects of metformin on creatinine solution were compared between standard and mixed methods. When normalized to conditions without metformin, the standard method exhibited significantly lower maximum reaction speed than the mixed method (Fig. 6A). Similarly, the final reaction speed and the ratio of the final to the maximum reaction speed were significantly higher in the standard method than in the mixed method (Fig. 6B and C). Although there was no significant difference, measured value was lower in standard method than in mixed method (Fig. 6D). These results suggest that the pre-incubation step in the standard method enhanced the inhibitory effect of metformin.

## 4 Discussions

Our initial findings revealed a spurious decrease in urinary creatinine levels in patients receiving metformin therapy. Given the rising global prevalence of diabetes, a 6.1 % age-standardized prevalence [1], and the widespread use of metformin as a first-line therapy since its development in the 1950s [2,3,22], the number of patients currently prescribed this drug is likely substantial. Furthermore, with the increasing adoption of enzymatic creatinine assays since their introduction in 1992, surpassing the traditional Jaffe method [9,10], metformin interference may have affected a considerable proportion of urinary creatinine measurements in patients with diabetes. This impact is likely to continue to increase in the future.

Metformin is primarily excreted in an unchanged form in urine, exhibiting a plasma elimination half-life of 4–9 h [3,5]. The reported urinary metformin concentrations range from 0 to 600 mg/dL [6,7], and our findings confirm that even 100 mg/dL significantly reduces creatinine levels. Accurate creatinine measurement is essential as it serves as a crucial reference for urine concentration and influences the assessment of various urinary biomarkers. The metformin-induced spurious decrease in creatinine levels may particularly affect urinary albumin measurements, which are critical for the early detection and monitoring of chronic kidney disease and diabetic nephropathy [8,23,24]. While the urinary albumin/creatinine ratio (ACR) is a key metric in these evaluations, harmonization of ACR determination remains a challenge, and standardization of albumin measurements may be necessary [25,26]. The spurious low creatinine values caused by metformin can lead to falsely elevated ACR values, potentially resulting in an inaccurate assessment of kidney function. Furthermore, metformin has been investigated in various clinical studies involving newly diagnosed type 2 diabetes mellitus, cardiovascular events, and polycystic kidney disease [[27], [28], [29]], many of which utilized urinary biomarkers, including ACR. In addition, metformin has been used as standard treatment drug to compare with the new treatment [30,31]. Consequently, metformin interference may affect laboratory testing and clinical research. Given that the inhibitory effect of metformin depends on its concentration and not on creatinine concentration, spurious low creatinine values may occur at any time in patients receiving regular metformin therapy.

**Fig. 7:**
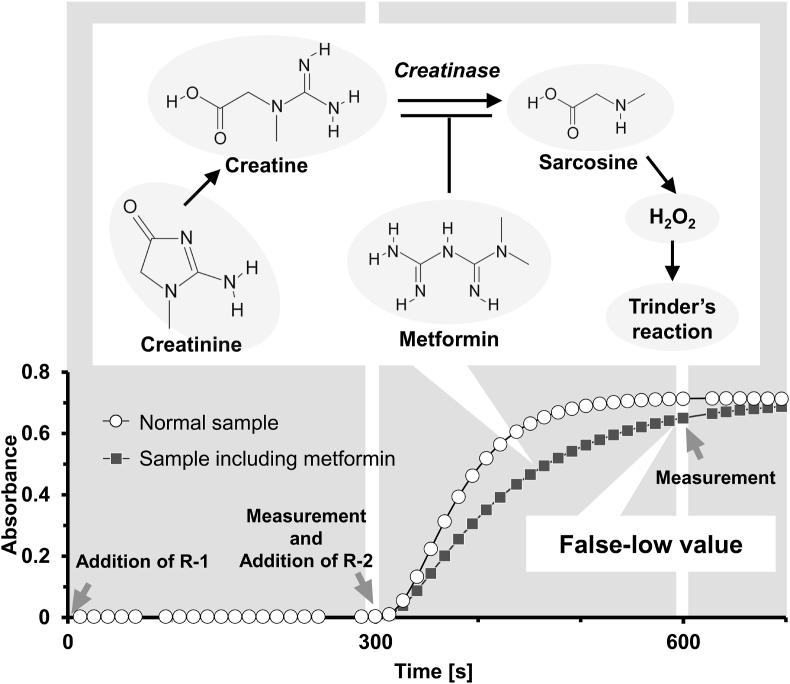
Scheme of metformin inhibition mechanism for urinary creatinine measurement. Metformin in urine samples forms a complex with creatinase in reagent 1, deleting endogenous creatine. After the addition of reagent 2, the creatinine in the urine sample was converted to creatine; however, creatinase combined with metformin delayed the series of creatinine measurement reactions. This phenomenon may lead to a false low urinary creatinine values.

Our initial findings revealed a spurious decrease in urinary creatinine levels in patients receiving metformin (Fig. 1). Metformin delayed the series of enzymatic reactions involved in creatinine measurement in a concentration-dependent manner (Fig. 2A), with an inhibitory effect correlating with metformin rather than creatinine (Table 1, Fig. 2B). Notably, the ratio of the final reaction speed to the maximum reaction speed appeared to be proportional to the degree of metformin interference, suggesting the potential for estimating creatinine values more accurately using reaction kinetic analysis. Measurements using the mixed method demonstrated that metformin interfered with both creatinine and creatine conversion, but not with sarcosine oxidation or hydrogen peroxide production (Fig. 3). Although the inhibitory effect of metformin on creatinine conversion was more pronounced than on creatine conversion, metformin did not inhibit creatininase activity (Fig. 4). This observation may be attributed to the gradual conversion of creatinine to creatine by creatininase, which potentiates the inhibitory effects of metformin at low creatine concentrations. Dixon plot analysis suggested the non-competitive inhibition of creatinase by metformin (Fig. 5). Although the lines on the Lineweaver-Burk plot intersected near the origin, this may be a consequence of analyzing the combined activities of three enzymes: creatinase, sarcosine oxidase, and peroxidase. Furthermore, the inhibitory effect of metformin was stronger in the standard method than in the mixed method, indicating that pre-incubation of the urine sample with reagent 1 enhanced the inhibition of creatinase by metformin (Fig. 6). These findings indicate that urinary creatinine values may be falsely low in patients receiving metformin therapy. The proposed mechanism of metformin interference is illustrated in Fig. 7.

Reaction kinetic analysis may be valuable in clinical laboratories for improving the accuracy of urinary creatinine measurements in patients taking metformin because ROC curve analysis indicated that the ratio of the final reaction speed to the maximum reaction speed could identify urine samples from patients taking metformin with sensitivity of 71 % and specificity of 87 % (Fig. 1D and E). The ratio of both speeds would be a potential indicator of the effects of metformin, and this metric is analogous to the two-point rate ratio used for prozone checks in certain assays. Therefore, if clinical laboratory scientists can identify samples suspected of metformin interference, retesting with a diluted sample could provide more accurate urinary creatinine values.

This study had a few limitations. First, our findings indicate that metformin inhibits creatinase, suggesting that the degree of metformin interference may vary across assays from different manufacturers. Therefore, clinical laboratories should evaluate the effects of metformin on creatinine measurements, including serum and urine samples, using specific reagents and methods. Second, the retrospective nature of this study, which employed a random sample selection, limited our ability to definitively establish a causal relationship between metformin use and urinary creatinine values. While reproducible experiments using aqueous solutions clarified the effects of metformin on the enzymatic assay, further investigation, including measurement of urinary metformin concentrations and comparison with alternative creatinine measurement methods (e.g., the Jaffe method, HPLC, other enzymatic assays, and liquid chromatography-mass spectrometry), is necessary to fully elucidate the effects of urinary metformin on creatinine measurements.

In conclusion, metformin interferes with urinary creatinine measurements, resulting in spuriously low urinary creatinine values. Given the non-competitive inhibition of creatinase by metformin, and the dependence of this effect on metformin rather than on creatinine, metformin may cause this artificial decrease even when actual creatinine levels are low. Monitoring the reaction curve may enable clinical laboratory scientists to obtain more accurate creatinine values by re-testing diluted specimens when metformin interference is suspected.

## CRediT authorship contribution statement

**Akira Yoshimoto:** Writing – original draft, Investigation, Formal analysis, Data curation, Conceptualization. **Yoshifumi Morita:** Writing – review & editing, Project administration, Formal analysis. **Yukio Kume:** Resources. **Naoyuki Yoshikawa:** Resources, Project administration. **Yoshikazu Ono:** Supervision. **Makoto Kurano:** Supervision. **Yutaka Yatomi:** Writing – review & editing, Supervision. **Ryunosuke Ohkawa:** Writing – review & editing, Supervision, Project administration, Funding acquisition.

## Data availability statement

Due to the nature of this research, the patient data from this study is not available, as participants did not consent to have their data shared publicly. However, the in vitro data supporting the findings of this study are available from the corresponding author (Akira Yoshimoto, Graduate School of Medical and Dental Sciences, Institute of Science Tokyo, yoshimoto.akira@tmd.ac.jp) upon reasonable request.

## Research funding

This study was supported by the Project research fund 2020–2021 from Japan Society of Clinical Chemistry Kanto Branch.

## Declaration of competing interest

The authors declare that they have no known competing financial interests or personal relationships that could have appeared to influence the work reported in this paper.

## Appendix A Supplementary data

Supplementary Fig. 1. The principle and procedure of creatinine measurement. After endogenous creatine is eliminated in 1st reaction, creatinine is converted into blue pigment by the addition of reagent 2. Creatinine measurement value is calculated from the difference in absorbance at 600 nm after the 1st and 2nd reaction.
