# Supplementary material for: Metformin interferes with urinary creatinine measurement using enzymatic method
Source: Biochem Biophys Rep. 2025 Sep 15;44:102264. doi: 10.1016/j.bbrep.2025.102264 (PMC12465047; doi:10.1016/j.bbrep.2025.102264)
Supplement: Multimedia component 1 [file mmc1.pptx]

## Slide 1
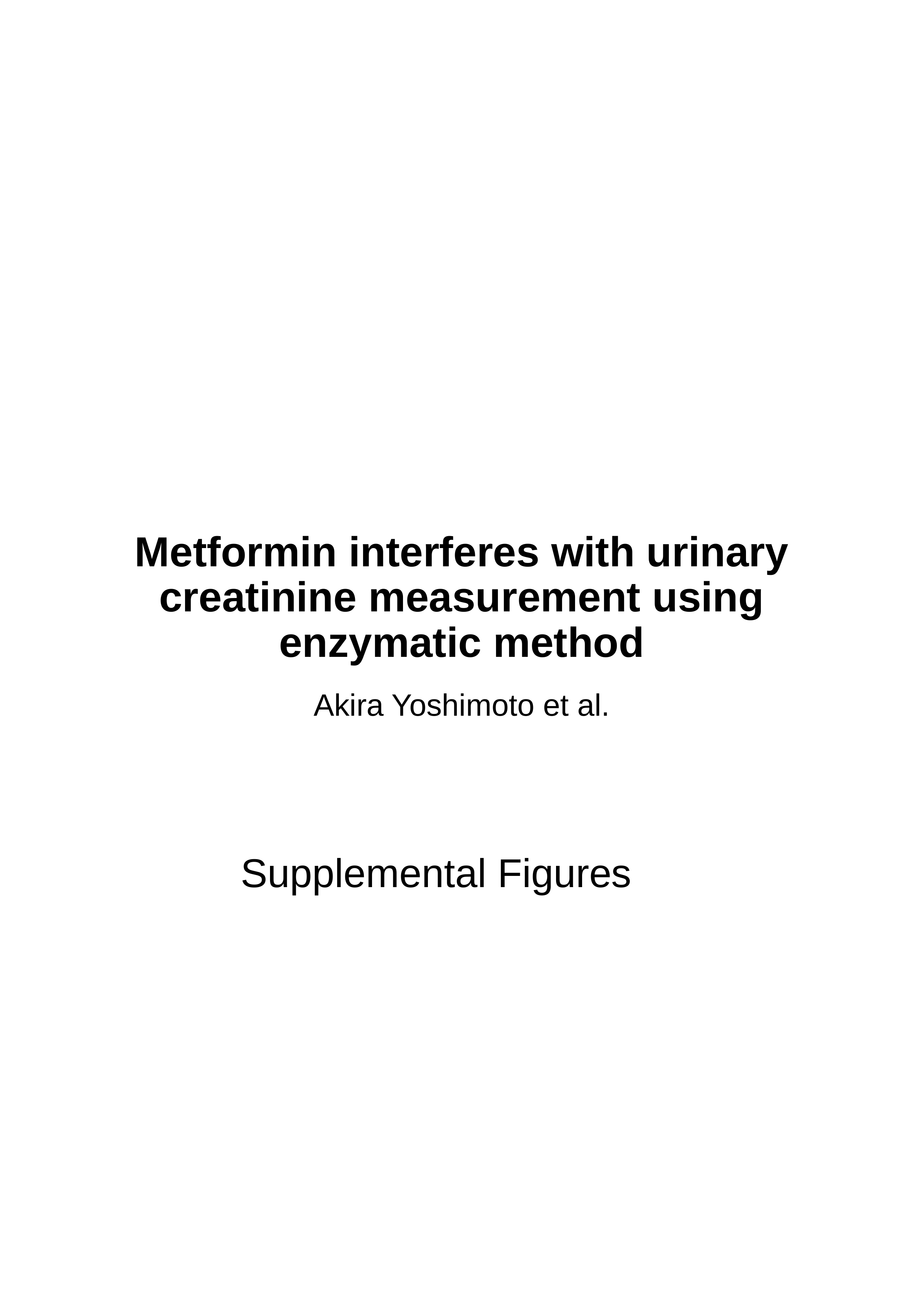

# Metformin interferes with urinary creatinine measurement using enzymatic method
Akira Yoshimoto et al.
Supplemental Figures

## Slide 2
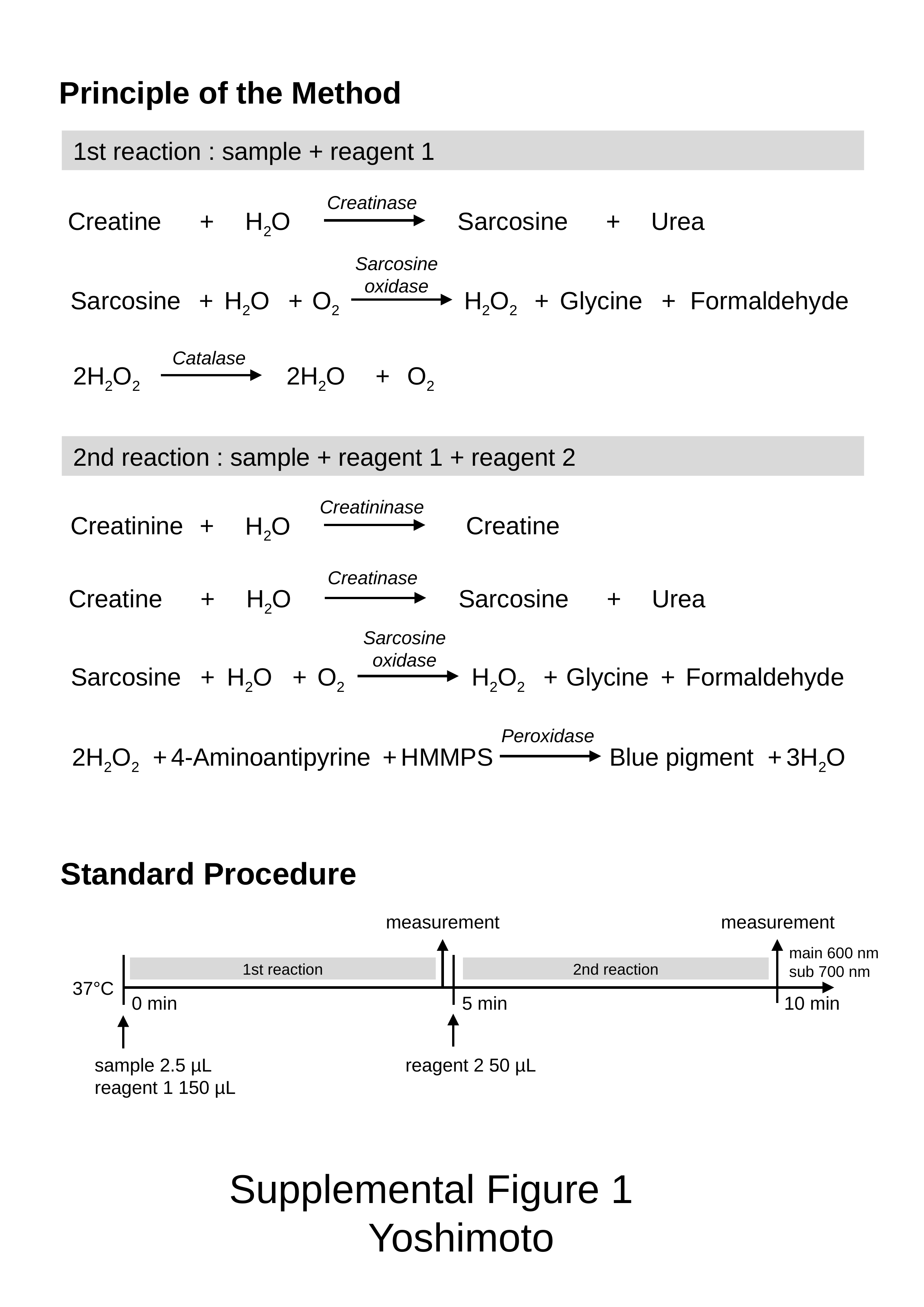

Principle of the Method
1st reaction : sample + reagent 1
Creatinase
Creatine
+
H2O
Sarcosine
+
Urea
Sarcosine oxidase
Sarcosine
+
H2O
+
O2
H2O2
+
Glycine
+
Formaldehyde
Catalase
2H2O2
2H2O
+
O2
2nd reaction : sample + reagent 1 + reagent 2
Creatininase
Creatinine
+
H2O
Creatine
Creatinase
Creatine
+
H2O
Sarcosine
+
Urea
Sarcosine oxidase
Sarcosine
+
H2O
+
O2
H2O2
+
Glycine
+
Formaldehyde
Peroxidase
2H2O2
+
4-Aminoantipyrine
+
HMMPS
Blue pigment
+
3H2O
Standard Procedure
measurement
measurement
main 600 nm
sub 700 nm
1st reaction
2nd reaction
37°C
0 min
5 min
10 min
sample 2.5 µL
reagent 1 150 µL
reagent 2 50 µL
Supplemental Figure 1　Yoshimoto
