# Supplementary material for: Metformin interferes with urinary creatinine measurement using enzymatic method
Source: Biochem Biophys Rep. 2025 Sep 15;44:102264. doi: 10.1016/j.bbrep.2025.102264 (PMC12465047; doi:10.1016/j.bbrep.2025.102264)
Supplement: Multimedia component 2 [file mmc2.docx]

STROBE Statement—checklist of items that should be included in reports of observational studies

|  | Item No. | Recommendation | Page  No. | Relevant text from manuscript |
| --- | --- | --- | --- | --- |
| **Title and abstract** | 1 | (*a*) Indicate the study’s design with a commonly used term in the title or the abstract | Abstract file  Page 1 | First, the reaction curves of urinary creatinine measurements from 328 patients were analyzed, focusing on the maximum reaction speed, final reaction speed, and their ratio. Next, the reaction curves of the creatinine solution with and without metformin were analyzed. To elucidate the mechanism of metformin interference, solutions of creatinine, creatine, sarcosine, and hydrogen peroxide with and without metformin were analyzed using a one-reagent measurement in which reagents 1 and 2 were pre-mixed. |
|  |  | (*b*) Provide in the abstract an informative and balanced summary of what was done and what was found | Abstract file  Page 1-2 | As the results, in the 84 patients taking metformin, the maximum reaction speed significantly decreased, whereas the final reaction speed and the ratio of the two speeds significantly increased (p < 0.001). Notably, the area under the curve of the ratio of the two speeds was 0.84 (95% confidence interval: 0.78-0.89) for detecting metformin use, suggesting that metformin inhibits the series of reactions involved in creatinine measurement and that reaction curve analysis can identify metformin use. Similar results were obtained in experiments using metformin-containing creatinine solutions. Metformin did not interfere with the reactions involving sarcosine or hydrogen peroxide but did interfere with those involving creatinine and creatine, indicating that metformin inhibits creatinase activity.  In conclusion, metformin inhibits reactions involved in urinary creatinine measurement in urine samples, potentially leading to falsely low urinary creatinine values and an inaccurate assessment of kidney function in patients with diabetes. |
| Introduction | | | |  |
| Background/rationale | 2 | Explain the scientific background and rationale for the investigation being reported | Page 2 | Autoanalyzers for biochemistry generate reaction curves for each specimen, enabling calculation of the measured values. Analyzing these reaction curves can be valuable for detecting potential interferences, such as M-proteins in bilirubin, urea, and high-density lipoprotein cholesterol measurements and homogentisic acid in creatinine measurement [13-16]. During routine laboratory testing, unusual reaction curves were observed in the creatinine assay of urine specimens from patients with diabetes receiving metformin therapy. Given the structural similarity between metformin and creatine, we hypothesized that metformin interferes with enzymatic creatinine measurements. To the best of our knowledge, this potential interference has not been reported previously. |
| Objectives | 3 | State specific objectives, including any prespecified hypotheses | Page 2 | we aimed to investigate the effects of metformin on urinary creatinine levels. |
| Methods | | | |  |
| Study design | 4 | Present key elements of study design early in the paper | Page 4 | Kinetic parameters, such as the maximum reaction speed, final reaction speed, and their ratio, were calculated from the reaction curves. A receiver operating characteristic (ROC) curve was generated using the ratio of the final reaction speed to the maximum reaction speed to determine whether patients took metformin or not. |
| Setting | 5 | Describe the setting, locations, and relevant dates, including periods of recruitment, exposure, follow-up, and data collection | Page 3 | Inpatients and outpatients at the University of Tokyo Hospital between July and September 2020 were enrolled. Inclusion criterion required that patients had undergone all of the following clinical laboratory tests on the same day: blood tests including albumin, blood urea nitrogen (BUN), creatinine, aspartate aminotransferase (AST), alanine aminotransferase (ALT), total cholesterol, triglycerides, blood glucose, and hemoglobin A1c, as well as urinary tests including urinary creatinine, albumin/total protein, and dipstick tests. Patients with urinary creatinine levels greater than 250 mg/dL, which was upper limit of linearity, were excluded. A total of 328 patients were therefore enrolled in the analysis. |
| Participants | 6 | (*a*) *Cohort study*—Give the eligibility criteria, and the sources and methods of selection of participants. Describe methods of follow-up  *Case-control study*—Give the eligibility criteria, and the sources and methods of case ascertainment and control selection. Give the rationale for the choice of cases and controls  *Cross-sectional study*—Give the eligibility criteria, and the sources and methods of selection of participants | Page 3 | Inpatients and outpatients at the University of Tokyo Hospital between July and September 2020 were enrolled. Inclusion criterion required that patients had undergone all of the following clinical laboratory tests on the same day: blood tests including albumin, blood urea nitrogen (BUN), creatinine, aspartate aminotransferase (AST), alanine aminotransferase (ALT), total cholesterol, triglycerides, blood glucose, and hemoglobin A1c, as well as urinary tests including urinary creatinine, albumin/total protein, and dipstick tests. Patients with urinary creatinine levels greater than 250 mg/dL, which was upper limit of linearity, were excluded. A total of 328 patients were therefore enrolled in the analysis. Patients with urinary creatinine levels greater than 250 mg/dL were excluded based on our institutional validation tests, which confirmed this value as the upper limit of linearity. Hence, 328 patients were enrolled in the analysis. |
|  |  | (*b*) *Cohort study*—For matched studies, give matching criteria and number of exposed and unexposed  *Case-control study*—For matched studies, give matching criteria and the number of controls per case | Not applicable | Not applicable |
| Variables | 7 | Clearly define all outcomes, exposures, predictors, potential confounders, and effect modifiers. Give diagnostic criteria, if applicable | Not applicable | Not applicable |
| Data sources/ measurement | 8* | For each variable of interest, give sources of data and details of methods of assessment (measurement). Describe comparability of assessment methods if there is more than one group | Page 4 | Kinetic parameters, such as the maximum reaction speed, final reaction speed, and their ratio, were calculated from the reaction curves. |
| Bias | 9 | Describe any efforts to address potential sources of bias | Not applicable | Not applicable |
| Study size | 10 | Explain how the study size was arrived at | Page 7 | ROC curve analysis was performed to determine the optimal cut-off value for identifying patients receiving metformin therapy based on their medical records. The statistical power was 100%, which was calculated from the patient numbers with and without metformin treatment, the area under the curve (AUC) value, and significant level 5%. |

Continued on next page

| Quantitative variables | 11 | Explain how quantitative variables were handled in the analyses. If applicable, describe which groupings were chosen and why | Page 4 | A receiver operating characteristic (ROC) curve was generated using the ratio of the final reaction speed to the maximum reaction speed to determine whether patients took metformin or not. |
| --- | --- | --- | --- | --- |
| Statistical methods | 12 | (*a*) Describe all statistical methods, including those used to control for confounding | Page 7 | Wilcoxon rank sum test was used to compare the reaction rates between patients taking and not taking metformin. ROC curve analysis was performed to determine the optimal cut-off value for identifying patients receiving metformin therapy based on their medical records. |
|  |  | (*b*) Describe any methods used to examine subgroups and interactions | Not applicable | Not applicable |
|  |  | (*c*) Explain how missing data were addressed | Not applicable | Not applicable |
|  |  | (*d*) *Cohort study*—If applicable, explain how loss to follow-up was addressed  *Case-control study*—If applicable, explain how matching of cases and controls was addressed  *Cross-sectional study*—If applicable, describe analytical methods taking account of sampling strategy | Not applicable | Not applicable |
|  |  | (*e*) Describe any sensitivity analyses | Page 7 | The statistical power was 100%, which was calculated from the patient numbers with and without metformin treatment, the area under the curve (AUC) value, and significant level 5%. |
| Results | | | | |
| Participants | 13* | (a) Report numbers of individuals at each stage of study—eg numbers potentially eligible, examined for eligibility, confirmed eligible, included in the study, completing follow-up, and analysed | Page 8 | Of the 328 patients whose urinary specimens were tested in routine laboratory tests, 213 had type 2 DM with or without metformin therapy (n = 84 and 129, respectively) (Supplemental Table 1). |
|  |  | (b) Give reasons for non-participation at each stage | Page 3 | Patients with urinary creatinine levels greater than 250 mg/dL, which was upper limit of linearity, were excluded. |
|  |  | (c) Consider use of a flow diagram | Not applicable | Not applicable |
| Descriptive data | 14* | (a) Give characteristics of study participants (eg demographic, clinical, social) and information on exposures and potential confounders | Page 8 | Of the 328 patients whose urinary specimens were tested in routine laboratory tests, 213 had type 2 DM with or without metformin therapy (n = 84 and 129, respectively) (Supplemental Table 1). Patients with taking metformin showed almost same mean age; however, they seemed to be lower serum creatinine level than patients without taking metformin. In patients group taking metformin, GFR stage of almost patients were relatively low and patients group without metformin included relatively lower kidney function such as G4 and G5 stages than patients group with metformin. |
|  |  | (b) Indicate number of participants with missing data for each variable of interest | Not applicable | Not applicable |
|  |  | (c) *Cohort study*—Summarise follow-up time (eg, average and total amount) | Not applicable | Not applicable |
| Outcome data | 15* | *Cohort study*—Report numbers of outcome events or summary measures over time | Not applicable | Not applicable |
|  |  | *Case-control study—*Report numbers in each exposure category, or summary measures of exposure | Not applicable | Not applicable |
|  |  | *Cross-sectional study—*Report numbers of outcome events or summary measures | *Page 9* | *The ROC curve analysis using the ratio of both speeds revealed that this ratio effectively discriminated between patients taking and not taking metformin, with an AUC of 0.84 (95% confidence interval, 0.78–0.89) (Fig. 1E). An optimal cutoff value of 0.87% yielded a sensitivity of 71% and specificity of 87% for identifying patients on metformin therapy.* |
| Main results | 16 | (*a*) Give unadjusted estimates and, if applicable, confounder-adjusted estimates and their precision (eg, 95% confidence interval). Make clear which confounders were adjusted for and why they were included | Not applicable | Not applicable |
|  |  | (*b*) Report category boundaries when continuous variables were categorized | Not applicable | Not applicable |
|  |  | (*c*) If relevant, consider translating estimates of relative risk into absolute risk for a meaningful time period | Not applicable | Not applicable |

Continued on next page

| Other analyses | 17 | Report other analyses done—eg analyses of subgroups and interactions, and sensitivity analyses | *Page 9* | *The ROC curve analysis using the ratio of both speeds revealed that this ratio effectively discriminated between patients taking and not taking metformin, with an AUC of 0.84 (95% confidence interval, 0.78–0.89) (Fig. 1E). An optimal cutoff value of 0.87% yielded a sensitivity of 71% and specificity of 87% for identifying patients on metformin therapy.* |
| --- | --- | --- | --- | --- |
| Discussion | | | | |
| Key results | 18 | Summarise key results with reference to study objectives | Page14 | Reaction kinetic analysis may be valuable in clinical laboratories for improving the accuracy of urinary creatinine measurements in patients taking metformin because ROC curve analysis indicated that the ratio of the final reaction speed to the maximum reaction speed could identify urine samples from patients taking metformin with sensitivity of 71% and specificity of 87% (Fig. 1D, E). The ratio of both speeds would be a potential indicator of the effects of metformin, and this metric is analogous to the two-point rate ratio used for prozone checks in certain assays. |
| Limitations | 19 | Discuss limitations of the study, taking into account sources of potential bias or imprecision. Discuss both direction and magnitude of any potential bias | Page 14 | the retrospective nature of this study, which employed a random sample selection, limited our ability to definitively establish a causal relationship between metformin use and urinary creatinine values. |
| Interpretation | 20 | Give a cautious overall interpretation of results considering objectives, limitations, multiplicity of analyses, results from similar studies, and other relevant evidence | Page 15 | In conclusion, metformin interferes with urinary creatinine measurements, resulting in spuriously low urinary creatinine values. Given the non-competitive inhibition of creatinase by metformin, and the dependence of this effect on metformin rather than on creatinine, metformin may cause this artificial decrease even when actual creatinine levels are low. Monitoring the reaction curve may enable clinical laboratory scientists to obtain more accurate creatinine values by re-testing diluted specimens when metformin interference is suspected. |
| Generalisability | 21 | Discuss the generalisability (external validity) of the study results | Page 14 | our findings indicate that metformin inhibits creatinase, suggesting that the degree of metformin interference may vary across assays from different manufacturers. Therefore, clinical laboratories should evaluate the effects of metformin on creatinine measurements, including serum and urine samples, using specific reagents and methods. |
| Other information | |  | | |
| Funding | 22 | Give the source of funding and the role of the funders for the present study and, if applicable, for the original study on which the present article is based | Page 16 | **Research funding**  This study was supported by the Project research fund 2020-2021 from the Japan Society of Clinical Chemistry, Kanto Branch. |

*Give information separately for cases and controls in case-control studies and, if applicable, for exposed and unexposed groups in cohort and cross-sectional studies.

**Note:** An Explanation and Elaboration article discusses each checklist item and gives methodological background and published examples of transparent reporting. The STROBE checklist is best used in conjunction with this article (freely available on the Web sites of PLoS Medicine at http://www.plosmedicine.org/, Annals of Internal Medicine at http://www.annals.org/, and Epidemiology at http://www.epidem.com/). Information on the STROBE Initiative is available at www.strobe-statement.org.
