# Supplementary material for: Metformin interferes with urinary creatinine measurement using enzymatic method
Source: Biochem Biophys Rep. 2025 Sep 15;44:102264. doi: 10.1016/j.bbrep.2025.102264 (PMC12465047; doi:10.1016/j.bbrep.2025.102264)
Supplement: Multimedia component 3 [file mmc3.docx]

**Metformin interference urinary creatinine measurement using enzymatic method**

Supplemental Table

Akira Yoshimoto^1,2^, Yoshifumi Morita^1^, Yukio Kume^1^, Naoyuki Yoshikawa^1^, Yoshikazu Ono^1^, Ryunosuke Ohkawa^2^, Makoto Kurano^1,3^ and Yutaka Yatomi^1,3,4^

^1^ Department of Clinical Laboratory, The University of Tokyo Hospital, Tokyo, Japan

^2^ Clinical Bioanalysis and Molecular Biology, Graduate School of Medical and Dental Sciences, Institute of Science Tokyo, Tokyo, Japan.

^3^ Department of Clinical Laboratory Medicine, Graduate School of Medicine, The University of Tokyo, Tokyo, Japan

^4^ Graduate School, International University of Health and Welfare, Tokyo, Japan

Supplemental table 1. Patients data

|  | Metformin(+) | | | Metformin(-) | | |
| --- | --- | --- | --- | --- | --- | --- |
| n |  | 84 |  |  | 244 |  |
| Male(n, %) | 57 |  | 68% | 141 |  | 58% |
| Age(years) | 65 | ± | 12 | 65 | ± | 16 |
| BMI(kg/m^2^) | 27 | ± | 4 | 24 | ± | 5 |
| Blood test |  |  |  |  |  |  |
| Albumin(g/dL) | 4.2 | ± | 0.3 | 4.0 | ± | 0.4 |
| BUN(mg/dL) | 15.9 | ± | 4.3 | 25.8 | ± | 18.2 |
| CRE(mg/dL) | 0.87 | ± | 0.26 | 1.60 | ± | 1.40 |
| AST(U/L) | 26 | ± | 13 | 23 | ± | 11 |
| ALT(U/L) | 27 | ± | 17 | 22 | ± | 18 |
| TC(mg/dL) | 177 | ± | 33 | 192 | ± | 38 |
| TG(mg/dL) | 167 | ± | 120 | 160 | ± | 151 |
| BG(mg/dL) | 149 | ± | 43 | 130 | ± | 48 |
| HbA1c(%) | 7.4 | ± | 1.1 | 6.5 | ± | 1.1 |
| Albuminuria/proteinuria categories |  |  |  |  |  |  |
| A1(n, %) | 67 |  | 80% | 119 |  | 49% |
| A2(n, %) | 8 |  | 10% | 50 |  | 20% |
| A3(n, %) | 9 |  | 11% | 75 |  | 31% |
| GFR stages |  |  |  |  |  |  |
| G1(n, %) | 14 |  | 17% | 19 |  | 8% |
| G2(n, %) | 36 |  | 43% | 59 |  | 24% |
| G3a(n, %) | 28 |  | 33% | 44 |  | 18% |
| G3b(n, %) | 6 |  | 7% | 51 |  | 21% |
| G4(n, %) | 0 |  | 0% | 46 |  | 19% |
| G5(n, %) | 0 |  | 0% | 25 |  | 10% |
| Urinary strip test |  |  |  |  |  |  |
| Specific gravity | 1.022 | ± | 0.009 | 1.015 | ± | 0.007 |
| pH | 5.7 | ± | 0.8 | 5.8 | ± | 0.7 |
| Protein (more than ±)(n, %) | 33 |  | 39% | 131 |  | 54% |
| Glucose (more than ±)(n, %) | 46 |  | 55% | 64 |  | 26% |
| Blood (more than ±)(n, %) | 13 |  | 15% | 165 |  | 68% |
| White blood cells (more than ±)(n, %) | 16 |  | 19% | 41 |  | 17% |

BUN, blood urea nitrogen; CRE, creatinine; AST, aspartate aminotransferase; ALT, alanine aminotransferase; TC, total cholesterol; TG, triglycerides; BG, blood glucose; HbA1c, hemoglobin A1c.

Albuminuria/proteinuria categories was classified base on the value of urinary albumin (A1: less than 30 mg/g creatinine, A2: 30~299 mg/g creatinine, A3: more than 300 mg/g creatinine) or the value of urinary protein (A1: less than 0.15 g/g creatinine, A2: 0.15~0.49 g/g creatinine, A3: more than 0.5 g/g creatinine), respectively

GFR stages was classified based on eGFR value (G1: more than 90 ml/min/1.73m^2^, G2: 60~89 ml/min/1.73m^2^, G3a: 45~59 ml/min/1.73m^2^, G3b: 30~44 ml/min/1.73m^2^, G4: 15~29 ml/min/1.73m^2^, G5: less than 15 ml/min/1.73m^2^)

Supplemental table 2. Analysis of reaction curves in intermediate products of creatinine measurement

|  |  |  | **Creatinine** | | | **p** |  | **Creatine** | | | **p** |  | **Sarcosine** | | | **p** |  | **Hydroperoxide** | | | **p** |
| --- | --- | --- | --- | --- | --- | --- | --- | --- | --- | --- | --- | --- | --- | --- | --- | --- | --- | --- | --- | --- | --- |
| Maximum reaction speed(ΔABS/min) | | | | | | | | | | | | | | | | | | | | | |
| Metformin | 0 |  | 0.4106 | ± | 0.0010 |  |  | 0.4576 | ± | 0.0017 |  |  | 0.8561 | ± | 0.0013 |  |  | 3.9403 | ± | 0.0151 |  |
| (mg/mL) | 200 |  | 0.3201 | ± | 0.0022 | <0.001 |  | 0.4628 | ± | 0.0014 | <0.001 |  | 0.8570 | ± | 0.0016 | 0.0118 |  | 3.9597 | ± | 0.0155 | - |
|  | 500 |  | 0.2404 | ± | 0.0010 | <0.001 |  | 0.4780 | ± | 0.0002 | <0.050 |  | 0.8569 | ± | 0.0033 | 0.9513 |  | 3.9717 | ± | 0.0139 | - |
|  | 1000 |  | 0.1752 | ± | 0.0015 | <0.001 |  | 0.4996 | ± | 0.0030 | <0.001 |  | 0.8630 | ± | 0.0008 | 0.9680 |  | 3.9643 | ± | 0.0095 | - |
| Last reaction speed(ΔABS/min) | | | | | | | | | | | | | | | | | | | | | |
| Metformin | 0 |  | 0.0060 | ± | 0.0002 |  |  | 0.0033 | ± | 0.0007 |  |  | -0.0023 | ± | 0.0001 |  |  | -0.0002 | ± | 0.0004 |  |
| (mg/mL) | 200 |  | 0.0341 | ± | 0.0008 | <0.001 |  | 0.0155 | ± | 0.0006 | <0.001 |  | -0.0024 | ± | 0.0002 | - |  | -0.0004 | ± | 0.0007 | - |
|  | 500 |  | 0.0463 | ± | 0.0002 | <0.001 |  | 0.0141 | ± | 0.0008 | <0.001 |  | -0.0019 | ± | 0.0001 | - |  | -0.0002 | ± | 0.0003 | - |
|  | 1000 |  | 0.0438 | ± | 0.0010 | <0.001 |  | 0.0084 | ± | 0.0006 | <0.001 |  | -0.0027 | ± | 0.0009 | - |  | 0.0001 | ± | 0.0007 | - |
| The ratio of last reaction speed to maximum reaction speed(%) | | | | | | | | | | | | | | | | | | | | | |
| Metformin | 0 |  | 1.5 | ± | 0.0 |  |  | 0.7 | ± | 0.1 |  |  | -0.3 | ± | 0.0 |  |  | 0.0 | ± | 0.0 |  |
| (mg/mL) | 200 |  | 10.6 | ± | 0.3 | <0.001 |  | 3.4 | ± | 0.1 | <0.001 |  | -0.3 | ± | 0.0 | - |  | 0.0 | ± | 0.0 | - |
|  | 500 |  | 19.2 | ± | 0.1 | <0.001 |  | 3.0 | ± | 0.2 | <0.001 |  | -0.2 | ± | 0.0 | - |  | 0.0 | ± | 0.0 | - |
|  | 1000 |  | 25.0 | ± | 0.8 | <0.001 |  | 1.7 | ± | 0.1 | <0.001 |  | -0.3 | ± | 0.1 | - |  | 0.0 | ± | 0.0 | - |
| Measured value of creatinine (mg/dL) | | | | | | | | | | | | | | | | | | | | | |
| Metformin | 0 |  | 106.1 | ± | 0.2 |  |  | 106.1 | ± | 0.4 |  |  | 107.0 | ± | 0.4 |  |  | 127.1 | ± | 0.4 |  |
| (mg/mL) | 200 |  | 96.3 | ± | 0.2 | <0.001 |  | 101.6 | ± | 0.1 | <0.001 |  | 107.0 | ± | 0.4 | - |  | 127.6 | ± | 0.4 | - |
|  | 500 |  | 78.2 | ± | 0.1 | <0.001 |  | 97.6 | ± | 0.2 | <0.001 |  | 106.9 | ± | 0.3 | - |  | 127.9 | ± | 0.4 | - |
|  | 1000 |  | 58.5 | ± | 0.2 | <0.001 |  | 97.1 | ± | 0.5 | <0.001 |  | 107.3 | ± | 0.1 | - |  | 127.8 | ± | 0.4 | - |

Each p-value was calculated using the Tukey Kramar test vs. metformin 0 mg/dL.
p-value - : one-way analysis of variance (ANOVA) showed p-value > 0.05 and Tukey Kramar test were not performed
